# Battey’s Operation

**Published:** 1883-08

**Authors:** 


TRANSACTIONS OF THE OBSTETRICAL AND GYNÆCOLOGICAL SOCIETY OF WASHINGTON, D. C. Stated Meeting, held November 17, 1882.

Dr. Jos. Taber Johnson read a long and interesting paper, giving a successful case of

BATTEY'S OPERATION, occurring in his practice last August.

The case presents the following history: The case was first seen by Dr. Johnson, in consultation with Dr. J. R. Riley, in June, 1882. Miss M. was an unmarried lady, twenty-nine years of age, and gave a history of ovarian pain and convulsions reaching back to date of puberty. She had been under the care of many physicians in Pennsylvania, her home, but had never been relieved. She was constantly growing worse, and had become a great burden to herself and friends. Her mental faculties had lost their vigor, and she presented a facial appearance much resembling a confirmed epileptic. Her constant pain, increasing at menstrual periods into agony, finally culminating in spasms, during the days of the flow, had destroyed all pleasure in living, and she looked forward to an early death as her only relief. Her pain and eclampsia were aggravated by an unrelieved menstrual molimen. For several months last year she had no flow, and during that time grew rapidly worse.

Her pain was only controlled by large doses of morphia, chloral or alcohol. Life was unendurable without these remedies, and they obtained for her but temporary comfort.

After a careful examination, Dr. Johnson expressed the opinion that the only hope of cure was in the removal of the ovaries, and he fully explained the dangers attending the operation. Miss M. desired to have the operation performed at once, if it presented even a ray of hope. She much preferred to die than to live on in the pain she was enduring. Life had no charms for her. The price she was compelled to pay for its continuance robbed death of all its horrors; indeed, she welcomed its approach as the only means of escape from a condition worse than death to her. After various consultations with physicians, friends and relatives, it was finally determined to operate, and on the 17th of August, 1882, Dr. Johnson removed both ovaries and one Fallopian tube by the abdominal section, the patient being in a private room in the Providence Hospital, of which institution Dr. J. is gynæcologist. The patient made a good recovery, and has since been well. She has had no sign of menstrual flow, and scarcely a sign of convulsions, since leaving the hospital. Has had a number of momentary attacks, without becoming unconscious or falling. She is now cheerful, happy, very grateful, and a useful member of the household—caring for others, instead of, as heretofore, being constantly cared for.

The President requested Dr. King to open the discussion.

Dr. King said that he felt certain he did not know as much on the subject under discussion as Dr. Johnson, who deserved to be specially complimented for the manner in which he had handled his subject, and for the skill with which he had performed the operation, the operation being the first of the kind in this District, and at the same time successful. He had no personal experience with the operation, and hence had but little to say; in fact, after the careful, elaborate paper just read, there was very little left to be said. In a curious book by Dr. Bulwer, published in 1623, the castration of women was treated of, although the subject was not handled from a scientific standpoint, and it was doubtful whether the cases reported were reliable. One of the cases given was amusing. A man had castrated a woman, and was brought to trial for it. The justices were greatly troubled about it, because there was no law against castration of women, although there was one against castration of males. They finally solved the difficulty by hanging him for stealing apples from the woman. The old historians told us that the king of Lydia kept castrated women. Female castration was also referred to by Hesychius, Athenæus, Aristotle, Suidas, Galen and others. All this seemed to show that the spaying of women was not a modern practice, but Battey's operation was novel in this, that it was undertaken for beneficent and curative purposes. Battey, in his paper read before the International Congress in London, in 1881, had collected all the cases from 1872 to 1881, given by different operators, 218 in all, of which, however, 25 were "incomplete" operations. Of the total 218, there were 26 in which the element of epilepsy (either with or without other abnormalities) was present. Of these 26 epileptic cases, over two-thirds are said to have been relieved by the operation, the record being as follows: Died, 4; cured, 14; improved, 4; not improved, 2; not cured, 1; not stated, 1. The mortality of the whole 218 cases contained in Battey's list, of which 10 were his own operations, was 18 per cent. Dr. King said that he could not criticise either the operation or Dr. Johnson's case; the result showed that the doctor had done well. At the same time, the necessity for such an operation was a sad commentary on the boasted scientific knowledge of the age, an age so full of advances in pathology, hygiene, therapeutics, materia medica, etc., inasmuch as we had to come to it at last, and conclude that with all our knowledge, nothing remained but to castrate. It seemed that so far we had failed to understand the causes, pathology, therapeutics and hygiene of these troubles, so that nothing was left but surgical interference. And, above all, it was an operation not confined to the old, but practiced upon young women between twenty and thirty, and upon some even in their teens. They were unsexed so far as reproduction was concerned, and rendered barren for life. True, some of the patients in the incomplete cases had afterward married and borne children. The great danger lay in this, that the successes might cause it to be a hobby ridden too much, a thing that happened when the Cæsarean operation first became popular in France; then the operation was said to have become as common as blood-letting. The danger in Battey's operation was the temptation held out to relieve the symptoms at once by the brilliant operation of oöphorectomy, and we might easily go to extremes in this matter. Take cases of epilepsy; was it frequent to spay men for this trouble? Yet we know that many cases depended on sexual excitations, and we know that this could often be kept down by long-continued and large doses of bromide of potassium. He thought the treatment ought to be urged, that the bromide should at least be kept up for years, just as we did in male patients, before resorting to castration. The question had occurred to him, whether ligation of the uterine and spermatic arteries would not be equally effective. Still, there was, perhaps, danger in ligatures, and beside this, the collateral circulation might be re-established. The idea was not new with him. Simpson had proposed ligature of the ovary, although Dr. King did not quite understand whether he meant ligation in bulk or of the vessels. Bartlett had successfully ligated the ovaries and cured his patient. As to myoma being an indication for the operation, he did not think that it was set down that mere development or rapid growth of such a tumor was an indication, unless there was dangerous hæmorrhage. As to Battey's report, showing all the cases he could collect, it was but natural that a man with a new operation would look upon the bright side of the subject. Then, again, all the cases had occurred within the last ten years. What would these women be in twenty years? For, while it was true that the operation had led to no special results in animals, we could not tell what would finally happen in women.

Dr. Kleinschmidt said it had been his privilege to be present at the operation, and he had also the opportunity of seeing the patient since, having visited her on yesterday with Dr. Johnson. The change in her facial expression was remarkable, and he certainly would not have known the woman, although he had looked right in her face during the operation. Her face had lost the low, almost brutish expression which was so striking when he first saw her upon the operating-table; and when she entered the room yesterday she wore a pleasant smile, and appeared very cheerful. Upon inquiry, she informed them that all her former severe attacks had left her; that she had slight attacks, lasting a minute or so, at the time of her former menstrual discharges, and that the last but one menstrual period had passed off without any attack. She could now take care of herself, and was not obliged to have some one constantly watching her. Dr. K. said that while, as regarding a perfect relief from these nervous attacks, the operation might not be called a complete success, yet it certainly was successful in this, that it had converted severe epileptiform seizures, most of them lasting from four to six hours at a time, during which it required several persons to hold her, into attacks of what our French brethren called *petit mal*, passing off in a few minutes, or even seconds, and consisting simply in a temporary loss of consciousness.

Dr. King would add to what he had said, that, almost immediately after Dr. Battey had read his paper before the International Congress, Dr. Grailey Hewitt read a paper on cases of hystero-epilepsy due to flexions of the uterus, which were cured by the use of an appropriate pessary.

Dr. Busey inquired, How would the operation do in vaginismus?

Dr. Ashford had not heard Dr. Johnson's paper read. He was, however, present at the operation, and was struck by the brilliant and successful manner in which it was done. He did not at that time consider that it was a fair test in the case under discussion, and thought that, had it failed altogether in relieving the nervous symptoms, it would have been no proof against the propriety of operating in these cases. Although he advised it in this case, he yet had doubts as to its success, because it looked to him as if the brain was seriously affected, and hence he could not see how success could be looked for. Still, it was one of those rare cases where patient, friends, and medical attendants all were anxious to have something done for her relief, so that almost any procedure looking to that end was justified, after failure of all ordinary means. Practically, he knew nothing of the operation, and very little of its bibliography, but he thought that Dr. King's remarks as to the danger of its becoming too popular were pertinent. Thus, he had noticed, in looking over the journals, that during the last year or two, the indications for which the operation was advised had greatly increased in number. At first the indications were laid down in narrow limits; now the limits were becoming much more extensive, whether from the results obtained or from mere speculations. The history of the operation itself certainly did not give a wide range of indications for it. It appeared less indicated in hystero-epilepsy than in uterine growths, where the induction of the menopause was a good reason for its performance, as shown by the results obtained, for the growths had ceased to enlarge, even if they had not become actually reduced. He held that the indications were stronger in epilepsy induced by ovulation, than in cases where the attacks of epilepsy occurred more frequently. It was also indicated in cases of absence of the uterus and atresia vaginæ, where the appearance of the menses caused serious trouble. In some cases of his own, he had had the operation in mind in order to relieve the hæmorrhage due to uterine fibroids, and had advised the patient to have it done. In one of these cases, he forgot that he had first advised against it, and she was now in charge of Dr. J. T. Johnson. He did not know whether the doctor had relieved her by treatment. The great good of Dr. Johnson's paper was that it set us all to thinking over the operation, and the probable and possible indications for it.

Dr. Louis Mackall (by request) said that he had listened to and learned a great deal that was interesting from the paper, and expressed his thanks to the author for it. He felt that he was unable to add anything to the subject. No doubt the operation would prove a boon to humanity; still, it was a question in his mind whether all the diseased conditions for which the operation was undertaken were likely to be benefited by its performance. Hystero-epilepsy, as we knew, did not have its seat in the ovaries; the condition of the latter simply furnished the exciting causes for the attacks. The real trouble lay in the condition of the nervous system, which in some of its parts was in an unstable molecular state. He would also remind us, that we again and again met with cases of epilepsy where the seizures occurred day after day, and in greater and greater severity, until finally the intellect was all but destroyed, when the patients had a brutish look, and when all therapeutic means had been exhausted in vain. Such a patient would perhaps be sent West to some quack, and by and by we would hear that under the care of this man he was greatly improved, that this improvement lasted for months, until finally he returned looking well, and showing by his actions that he was in all respects vastly improved. This apparent recovery would go on for a time, and then, gradually, the man would fall back to his former condition. Then, very likely, another quack would be tried, and again improvement would take place. All of us, no doubt, knew of such cases and were surprised at the results. It was necessary, therefore, to wait some time before we could finally decide upon the success of this operation in any given case. It might be that by the removal of the ovaries a certain beneficial impression might be made upon the central nervous system, something similar to what we saw in some cases of trephining for epilepsy, where good results followed, even though no evidence of pressure on the brain was found. These remarks, however, he did not intend as a reflection upon the operation. There was one class of cases, not referred to, in which he deemed the operation perfectly justifiable, viz., a displaced ovary which caused intense suffering when bound down in Douglas' fossa. Here, as a rule, only one ovary need be removed, and by way of the vagina. There was one statement made by the doctor in regard to the vaginal operation, that it was less dangerous than the abdominal section. He had seen in a recent medical journal statements of a contrary character.

Dr. Ashford said that it was claimed that the results obtained by the vaginal method were not as good, but that the operation itself was less fatal than the abdominal section.

Dr. J. T. Johnson.—The mortality, as given, was 17 per cent. for the vaginal, and 24 per cent. for the abdominal operation.

Dr. Ashford drew attention to the condition of the ovaries in Dr. Johnson's case. In some cases they were normal, hence the name of "normal ovariotomy" given to the operation; but in most of the cases there was a diseased condition, and in these the success of the operation had been greatest; in other words, the disease here depended upon the abnormal condition of the ovaries.

Dr. W. W. Johnston stated, as a curious coincidence, that there were now in this room specimens of the first ovariotomy performed in Washington, by Dr. May, and of the first oöphorectomy performed here. In Dr. May's case the operation was easy and the patient recovered. The case had never been published.

Dr. Mackall inquired whether there were not among the cases reported some of displaced ovaries.

Dr. J. T. Johnson, in reply, stated that the operation had been done for the relief of that condition, and the suggestion was made to remove them by the less dangerous vaginal route. We could feel the organ in Douglas' fossa, and easily cut down upon it, while, if we attempted to reach it by laparotomy, we would be obliged to hunt for it in the pelvic fossa, and the adhesions might render the operation a very bloody one. In hernia of the ovary, the operation was also easy. In closing the discussion, he said he was indebted to the Society for the kind remarks made. He would never have undertaken the operation, had he not had the encouraging advice of his friends, all of whom urged it. He must thank his assistants at the operation itself. It was said that no one knew, in a case of this kind, what he would find upon opening the abdominal cavity; in his own case, fortunately, everything was easy, so much so that he was almost afraid it might turn his own head. When, in his paper, he had spoken of the difficulties of the operation as pointed out by Spencer Wells and others, he had no idea of lauding his own good fortune and success, for in his case all was perfectly plain sailing. The name "normal ovariotomy" would not apply here, for there were cysts in the ovaries, the broad ligament, and the Fallopian tube. Some of these, in time, might have enlarged and finally produced a large tumor. His patient had suffered for sixteen years. But even if it had only been known that these cysts existed, the operation would have been justifiable.—*Am. Journal of Obstetrics.*

